# Hair pyrrole adducts serve as biomarkers for peripheral nerve impairment induced by 2,5-hexanedione and *n*-hexane in rats

**DOI:** 10.1371/journal.pone.0209939

**Published:** 2018-12-31

**Authors:** Xianjie Li, Qiong Wang, Ming Li, Shuo Wang, Cuiqin Zhang, Keqin Xie

**Affiliations:** 1 Institute of Toxicology, School of Public Health, Shandong University, Jinan, Shandong Province, China; 2 School of Pharmaceutical, Liaocheng University, Liaocheng, Shandong Province, China; Federal University of Santa Catarina, BRAZIL

## Abstract

Pyrrole adducts are specific reaction products of 2,5-hexadione (2,5-HD) *in vivo* and are considered highly relevant to the pathogenesis of peripheral nerve impairments after exposure to *n*-hexane, though the exact mechanism remains unclear. In this study, 40 male Wistar rats were randomly divided into three experimental groups and one control group, in which all rat’s hair were shaved completely at the beginning. The rats in three experimental groups were treated with 2,5-HD by gavage at dosages of 100, 200 and 300 mg/kg per day respectively, six times per week for 8 weeks. Abnormality of gait and changes in the rota-rod latency were surveilled. Pyrrole adducts in hair, urine and serum of all rats were measured at the endpoint. Results showed that the increased pyrrole adducts in hair, urine and serum accumulated in dose-response relationship. Spearman’s correlation analysis between pyrrole adducts and gait scores showed that hair pyrrole adducts were highly relevant to the gait scores. Moreover, we treated rats with *n*-hexane and succeed to verify the results aforesaid. Further, multiply linear regression analysis showed that hair pyrrole adducts have higher partial correlation coefficients than these in serum and urine in both 2,5-HD and *n*-hexane treated models. Our findings draw the conclusion that the hair pyrrole adducts might serve as a promising biomarker of *n*-hexane induced peripheral neuropathy.

## Introduction

*N***-**Hexane (CH_3_(CH_2_)_4_CH_3_) is a straight chain saturated hydrocarbon obtained from certain petroleum fractions. It is widely used in the pharmaceutical and cosmetic industries and is a cleaning agent for textiles, furniture and leather products. Chronic exposure to *n*-hexane may produce important peripheral neuropathy in humans and experimental animals [1–3]. Clinical symptoms to *n*-hexane poisoning vary from slightly omit and dizziness to limbs hypoesthesia and lower extremity weakness, sometimes even paralyze [4–7].

*N*-Hexane is absorbed following inhalation, ingestion or by topical application to the skin, then distributed into the blood and transported to the liver. *N*-hexane is metabolized in the liver by sequential (*δ*-1)-oxidation to the 2,5-hexandione, which is widely considered to be the ultimate toxic metabolite [8]. However, it was then found that the carbonyl carbons of 2,5-HD represent electrophilic sites which are sequential attacked by nucleophilic groups containing lysine and serine. Therefore, 2,5-HD can be further metabolized to dimethylated pyrrole adducts [9–13]. Yin’s studies proved that he half-life time of 2,5-HD is about 2.5 hours, while the half-life time of pyrrole adducts is 22–35 hours [14]. Thus, pyrrole adducts can accumulate in organism alongside the chronic exposure. It is widely accepted that pyrrole adducts accumulation is responsible for the development of neuropathy induced by *n*-hexane [15–18]. The amount of pyrrole adducts in bio-samples are highly positively relevant to the degrees of impairment induced by 2,5-HD or *n*-hexane. Therefore, it is of paramount importance to monitor the accumulation of pyrrole adducts in organism. Nevertheless, the collection of nerve samples are impractical, thus occupational surveillance commonly use serum and urine samples to evaluate the statues of workers.

Kessler W [19] et al found that the pyrrole adducts could react with the Ehrlich’s reagent, showing unique fuchsia color with specific length of 526nm. That is, by measuring the absorption values, the concentrations of pyrrole adducts could be determined with spectrophotometry. Yin and Wang have determined the concentration of pyrrole adducts in serum, urine, sciatic nerves and elucidated there was a significant correlation between the peripheral neuropathy and pyrrole adducts [20–22]. Nevertheless, the measurements of pyrrole adducts in serum and urine might merely offer a transient level of exposure and might not be probable to determine long-time storage after exposure. Pyrrole adducts could be eliminated from organism completely 5 days later after last exposure, while cases had been reported that workers exposed to *n*-hexane occupationally revealed intoxication symptoms even three months off work. Moreover, the pyrrole adducts levels in urine and serum also drop drastically once the exposure terminated.

Hair samples have been used to evaluate chronic exposure to drugs or toxins over much longer periods of months to years. The growth of hair is highly structured, whose compact and organized structure make it possible for chemicals to aggregate and store for weeks or even months. Previously, Johnson DJ and Lack J et al have already detected pyrrole adducts in hair from rats treated with 2,5-hexadione by using spectrophotometric methods, but both of them failed to quantify the concentration of pyrrole adducts [23, 24].

Our study aimed to generate peripheral hinder limb paralyzed models by 2,5-HD administration to rats. Neuro-behavior tests were performed to evaluate the impairment degrees along the treatment. On the other hand, pyrrole adducts in hair, urine and serum were determined at the endpoint. Spearman’s correlation analyses were conducted to evaluate the relationship between the hair pyrrole adducts and those impairments. Then, models exposed to *n*-hexane were built to verify conclusion ahead. Further, partial correlation coefficients were calculated to verify whether hair pyrrole adducts have the potential to be a biomarker of *n*-hexane intoxication.

## Materials and methods

### Chemicals and reagents

Sodium hydrate, absolute ethanol, concentrate hydrochloric acid, guanidine chloride and *n*-hexane were purchased from Hongyan Reagent Factory (Tianjin, China). 2,5-hexanedione, 2,5-dimethylpyrrole, 4-dimethylaminobenzaidehyde (DAMB) and trypsin were purchased from Sigma-Aldrich Company (USA). BSA and BCA Protein Quantification Kit were purchased from Thermos Scientific Co. (USA). Detergents were purchased from nearby supermarket.

### Animal treatments

All animal experimental procedures and protocols of the study were approved by the Ethics Committee for Animal Experiments of Shandong University Institute of Preventive Medicine (Permit Number: 20111231) in accordance with the NIH Guide for Care and Use of Laboratory Animals.

Male Wistar rats, seven weeks old and weighing 210–220 g, were provided by Experiment Animal Center of Shandong University. All rats were accommodated with 22 ± 2 °C and 50 ± 10% relative humidity at animal room of a 12 h light / dark cycle, drinking water and commercial animal feed were available ad libitum. After the acclimatization period, 40 rats were divided into four groups randomly (n = 10 in each group), including one control group and three 2,5-HD experimental groups (100, 200 or 300 mg/ kg/ day, orally). Treated groups were administered with 2,5-HD dissolved in saline six times per week for consecutive eight weeks. Control group rats receive equal volume of saline vehicle.

As to the *n*-hexane model, 40 rats were randomly divided into control group and three *n*-hexane treated groups (1.0, 2.0 or 3.0 g/ kg/ day, orally), 10 of each group. Experimental groups rats were treated with *n*-hexane dissolved in corn oil six times per week for consecutive ten weeks. Control group rats receive equal volume of corn oil vehicle. At the endpoint, animals were sacrificed after anesthetized by urethane through haemospasia form the aorta abdominals.

### Neuro-behavior assessments

Gait scores of rats were determined at each weekend. Rats were placed in an open field and observed for 3 minutes. Following observation, a gait score was evaluated from 1 to 4, where 1 = a normal, unaffected gait; 2 = a slightly abnormal gait (hind limbs show uncoordinated placement, exaggerated or overcompensated movements, or are splayed slightly, walk on tiptops); 3 = moderately abnormal gait (obvious movement abnormalities characterized by markedly splaying hind limbs, ataxia, swaying, rocking, lurching, stumbling); 4 = severely abnormal gait (flat foot walk, hind legs flat on surface, crawling, or unable to support weight) [25, 26]. The assessment of gait scores was conducted blinded to treatment.

Rota-rod latency were measure using a Rota-rod equipment (ZS-ROM, Beijing Zhongshidichuang Technology and Development Co., Ltd, Beijing, China). According to Monville [27], all rats received a training before intoxication, which requires rats to stay on the equipment for at least 60 seconds at the velocity of 8 rpm. In formal test, the original velocity was set at 0 rpm and accelerated smoothly to 40 rpm within 200 seconds. The lengths of time that each animal stayed on the equipment was recorded as the latency to fall, registered automatically by a trip switch. Rota-rod latency tests were conducted per weekend.

### Pyrrole adducts assessments

When gait scores of rats in high dosage group all reached 4 point, separately 2,5-HD treated group at 8^th^ week while *n*-hexane treated group at 10^th^ week, all of the animals were sacrificed by haemospasia. Blood samples were collected form the aorta abdominals after anesthetized by urethane. After stewing for 2 hours, blood samples were centrifuged at 14000 g for 15 min at 4 °C, and serum samples were isolated and stored at -80 °C until measurement. Urine samples were collected by bladder stimulation before the sacrifice, after centrifuged at 14000 g for 15 min at 4 °C, the upper layer liquid were collected and stored as the serum samples aforesaid.

2.50 g of DMAB dissolved into 25 ml of concentrate hydrochloric acid, then metered the solution with a volumetric flask of 250 ml to prepare the Ehrlich’s reagent. According to Yin’s study [20], after unfreeze, 50 μl of each blood or urine sample, was added into a 96-well plate. Then 50 μl of guanidine hydrochloride (6 mol/l) and 50 μl of Ehrlich's reagent were added in turns. After that, absorption values were measured at the length of 526 nm with an automatic microplate reader (Infinite200PRO, TECAN INc, Switzer). The calculations were based on standard curve prepared with different concentrations of 2,5-dimethylpyrrole (2,5-DMP) (0–200 nmol/ml).

Rats were shaved off dorsal hair with an electronic razor ahead of treatment. Then hair samples were collected from the same part before the rats were sacrificed. Washed the hair samples completely with commercial detergent, defatted twice by absolute ethanol and dried the hair under room temperature. 75 mg of hair sample was added into 5 ml of 0.72 mol/l NaOH, water-bathing heated for 1 hour at 56°C. Then 200 μl of concentrated hydrochloride was used to adjust the pH to 8–9, and 0.2% trypsin solution was added to adjust volume to 10ml. After another 1 hour of heating in water-bath at 56°C, the digestion solutions were centrifuged the digestion at 14000 g for 10 minutes. 50 μl of supernatant from digestion solutions, 50 μl of guanidine hydrochloride (6 mol/l), 100 μl of absolute ethanol, 50 μl of Ehrlich's reagent were added into a 96-well plate in turns. Then absorption values were measured as foresaid. The calculations of working curve were conducted by co-digestion and measurement of different concentrations of 2,5-DMP and control samples (0–75.00 nmol/ml: 0, 3.00, 7.50, 11.25, 15.00, 30.00, 75.00 nmol/ml).

Afterwards, the internal and intergroup precision, standard recovery rate and limit of detection (LOD) were calculated. Firstly, pyrrole adducts of 5 hair samples of control group were measured as aforesaid, each sample digested repeated 5 times, then absorption values obtained were used to calculated the internal precision and intergroup precision. Afterwards, 2,5-DMP was added into hair solution of control group and constant volume to concentration of 3.00, 15.00 and 75.00 nmol/ml, then absorption values were measured and compared with each co-digestion standard concentration solution to calculate the 2,5-DMP standard recovery rate. Finally, LOD value was calculated through measuring the pyrrole adducts in control samples for 50 times. It was approved by calculations based on the standard deviation of the response (δ) and the slope (S) of the calibration curve at the levels approaching the limits according to equation LOD = 3.3 (δ/S)

The concentration of each sample digestion was determined using the 2,5-DMP working curve. After that, protein concentration quantification of each sample digestion solution was conducted by BCA protein quantification kit, then concentrations of pyrrole adducts in hair samples were adjusted using the protein concentrations.

### Data analysis

All data were expressed as the mean ± standard deviation. SPSS 19.0 software was used for statistical analysis. All data were analyzed using one-way analysis of variance (ANOVA). Spearman’s correlation analysis was performed, then multiple linear regression analyses were performed and partial correlation coefficients were calculated. The level of statistical significance was set at *P* < 0.05.

## Result

### Weight changes

**Fig 1:**
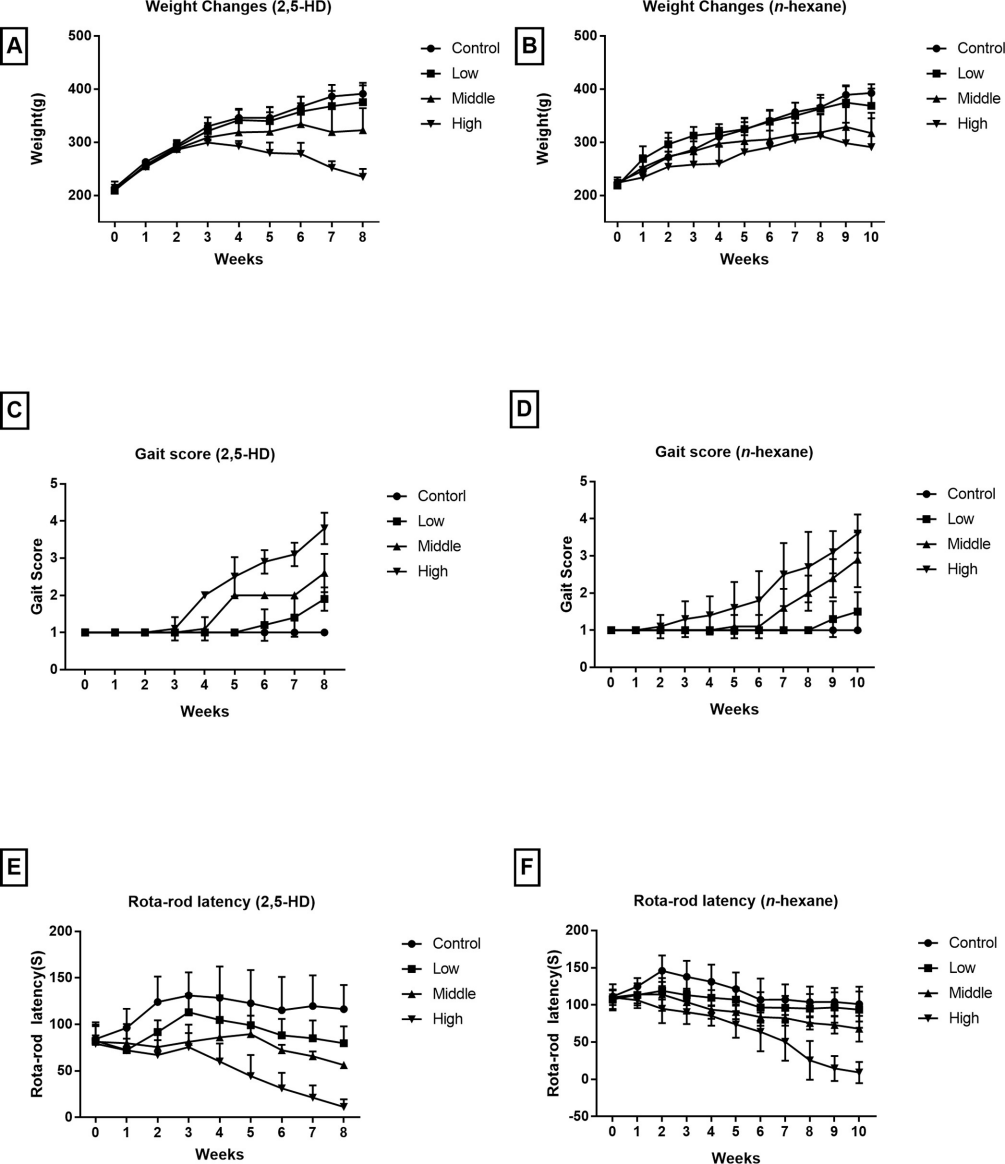
Weight and neurobehaviors. (A) Weight changes of rats treated with 2,5-HD. (B) Weight changes of rats treated with *n*-hexane. (C) Gait score changes of rats treated with 2,5-HD. (D) Gait score changes of rats treated with *n*-hexane. (E) Rota-rod latency changes of rats treated with 2,5-HD. (F) Rota-rod latency changes of rats treated with *n*-hexane.

As shown in Fig 1A & 1B, in 2,5-HD treated models, the weights of rats in high and middle dosage group began to decrease at the 3^rd^ and 6^th^ week, respectively. At the endpoint, the average weight of rats in low, middle and high groups were separately 96.0%, 82.5% and 60.1% of control. In *n*-hexane treated models, the weights of rats in high, middle and low dosage group began to decrease at the 8^th^, 9^th^ and 9^th^ week, respectively. At the endpoint, the average weight of rats in low, middle and high groups were separately 93.9%, 80.8% and 74.1% of control. Animals were in good conditions, no other symptoms underwent apart from the intoxication of 2,5-HD and *n*-hexane.

### Neuro-behavior assessments

Both *n*-hexane and 2,5-dihexadione induced significant peripheral neuropathy. By the end of third week, there were significance differences in scores between high dose group and the control group in both models treated with 2,5-HD and *n*-hexane (Fig 1C & 1D). By the end of treatment, gait scores of 2,5-HD treated groups were 1.9 ± 0.3, 2.6 ± 0.6 and 3.8 ± 0.4, showing significant differences contrasted with the control group (*P*<0.01). While the gait scores of the *n*-hexane treated group were 1.5 ± 0.5, 2.9 ± 0.7 and 3.6 ± 0.5, significant different from control group (*P*<0.01).

After the training, the latencies before treatment were measured, showing no significant difference (*P*>0.05). Monitoring results showed that administration of 2,5-HD and *n*-hexane shorten latencies severely (Fig 1E & 1F). As the rats grew, then the latencies of control groups reach a planet, while latencies of treated groups showed trends of decreases. At the endpoint, rats of high dosage groups could barely stand on the test machine.

### Pyrrole adducts assessments

**Fig 2:**
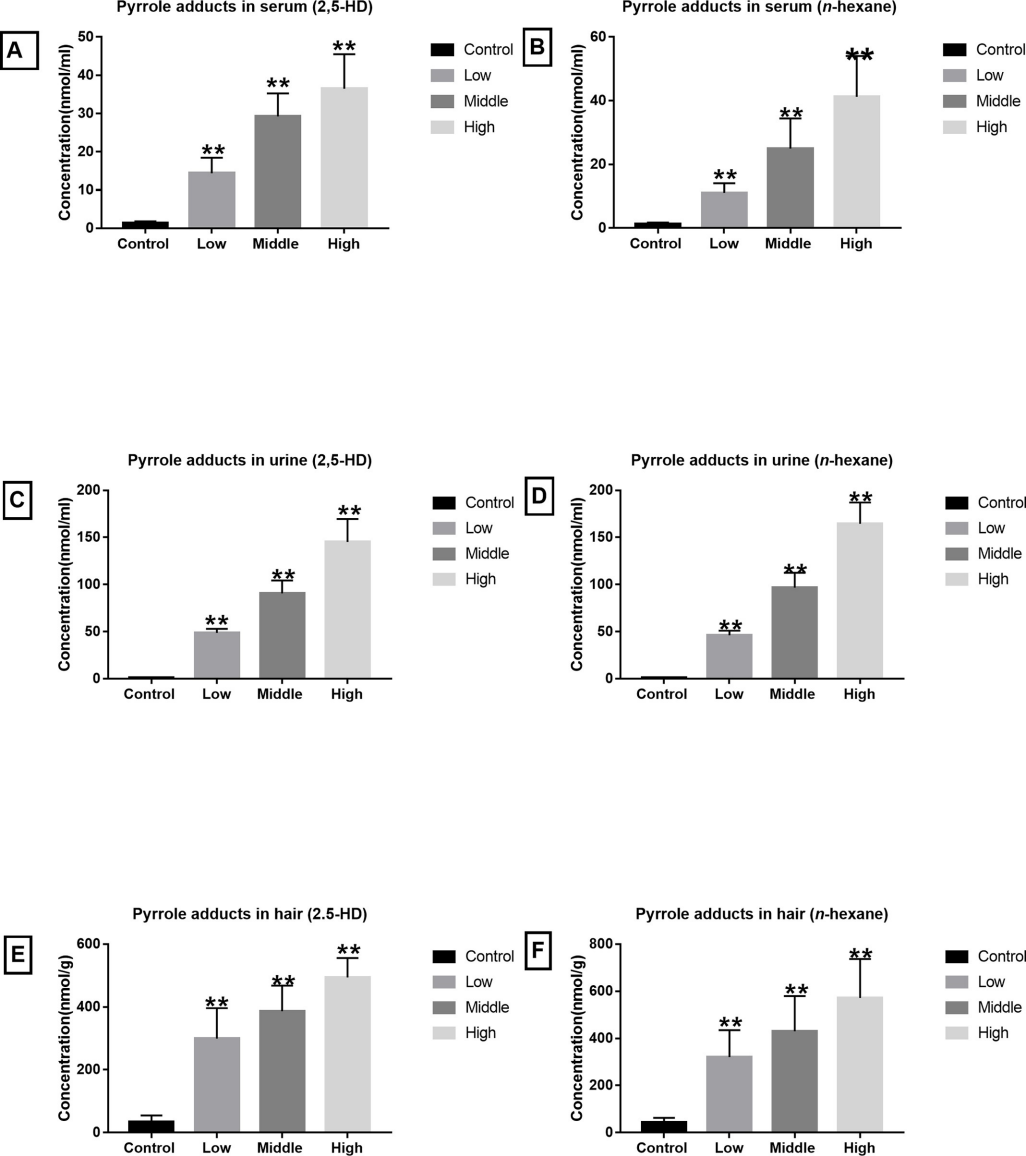
Pyrrole adducts accumulation. (A) Pyrrole adducts in serum of rats treated with 2,5-HD. (B) Pyrrole adducts in serum of rats treated with *n*-hexane. (C) Pyrrole adducts in urine of rats treated with 2,5-HD. (D) Pyrrole adducts in urine of rats treated with *n*-hexane. (E) Pyrrole adducts in hair of rats treated with 2,5-HD. (F) Pyrrole adducts in hair of rats treated with *n*-hexane. Compared with control group, ^**^P<0.01.

As shown in Fig 2, pyrrole adducts in serum, urine and hair at the endpoint all raised and showed significantly different from the control in both 2,5-HD and *n*-hexane treated models. Table 1 presented serum pyrrole adducts concentration at the endpoint. There were significant increases of pyrrole adducts concentrations in serum after administration of 2,5-HD and *n*-hexane, representing a dose-response relationship (*P*<0.01). Comparison between the models showed that the serum pyrrole adducts in low dosage groups of two models showed no significant differences, meanwhile, no differences appeared between the middle dosage groups and high dosage groups (*P*>0.05).

**Table 1:** Pyrrole adducts concentration on the endpoint.

	2,5-HD (nmol/ml)		*n*-hexane (nmol/ml)	
	Serum	Urine	Serum	Urine
Control	1.4 ± 0.4	1.2 ± 0.3	1.3 ± 0.4	1.1 ± 0.2
Low dosage	14.4 ± 4.0^**^	48.7 ± 4.4^**^	11.1 ± 3.0^**^	46.2 ± 4.8^**^
Middle dosage	29.3 ± 6.0^**^	90.7 ± 13.7^**^	25.0 ± 9.4^**^	96.7 ± 15.9^**^
High dosage	36.5 ± 9.0^**^	145.3 ± 24.2^**^	41.2 ± 12.8^**^	164.5 ± 22.5^**^

Compared with control group,

^**^P<0.01.

As shown in Table 1, significant increases of pyrrole adducts concentrations were observed in urine after administration of 2,5-HD and *n*-hexane, which showed a dose-response relationship (*P*<0.01). Still, no differences existed in the concentrations of urine pyrrole adducts within two models (*P*>0.05).

The working curve was calculated as foresaid by measuring optical density of a series of 2,5-DMP and blank hair co-digestion standard concentration solution, then the formula obtained: y = 0.0048x+0.0612, R^2^ = 0.9993, with linearity range between 0.00~75.00 nmol/ml. Then the LOD values was calculated of 0.049 nmol/ml, the internal and intergroup precision were both less than 10% (Table 2), meanwhile standard recovery rate was between 90.3%~107.2%, which means the methods was applicable (Table 3).

**Table 2:** Internal precision and intergroup precision of hair digestion.

NO.	Internal precision					Intergroup precision
	1	2	3	4	5	
1	0.0576	0.0585	0.0579	0.0579	0.0584	0.0581
2	0.0579	0.059	0.0577	0.0586	0.0649	0.0596
3	0.0555	0.0583	0.0589	0.0576	0.0613	0.0583
4	0.0594	0.0603	0.0609	0.0599	0.0706	0.0622
5	0.0595	0.0601	0.0606	0.0617	0.0716	0.0627
mean	0.0580	0.0592	0.0592	0.0591	0.0654	0.0602
SD	0.0016	0.0009	0.0015	0.0017	0.0057	0.0022
RSD	2.8	1.5	2.5	2.8	8.8	3.6

**Table 3:** 2.5-dimethylpyrrole standard recovery rate of hair digestion.

Concentration(nmol/ml)	1	2	3	4	5	mean	RSD
3.00	103.4	105.6	104.6	103.3	96.3	102.6	3.2
15.00	98.0	107.2	100.5	106.0	90.3	100.4	6.1
75.00	101.9	98.0	96.7	97.6	93.8	97.6	2.7

Then concentrations of hair samples at the endpoint were measured (Table 4), showing accumulation of pyrrole adducts in the hair.

**Table 4:** Pyrrole adducts concentration in hair on the endpoint.

	2,5-HD model (nmol/g.pro)	*n*-hexane model (nmol/g.pro)
Control	33.8 ± 20.8	42.9 ± 19.9
Low dosage	299.4 ± 96.6^**^	320.9 ± 114.1^**^
Middle dosage	386.9 ± 81.3^**^	430.2 ± 149.2^**^
High dosage	494.4 ± 61.6^**^	571.4 ± 165.8^**^

Compared with control group,

^**^P<0.01.

### Correlation analysis

**Fig 3:**
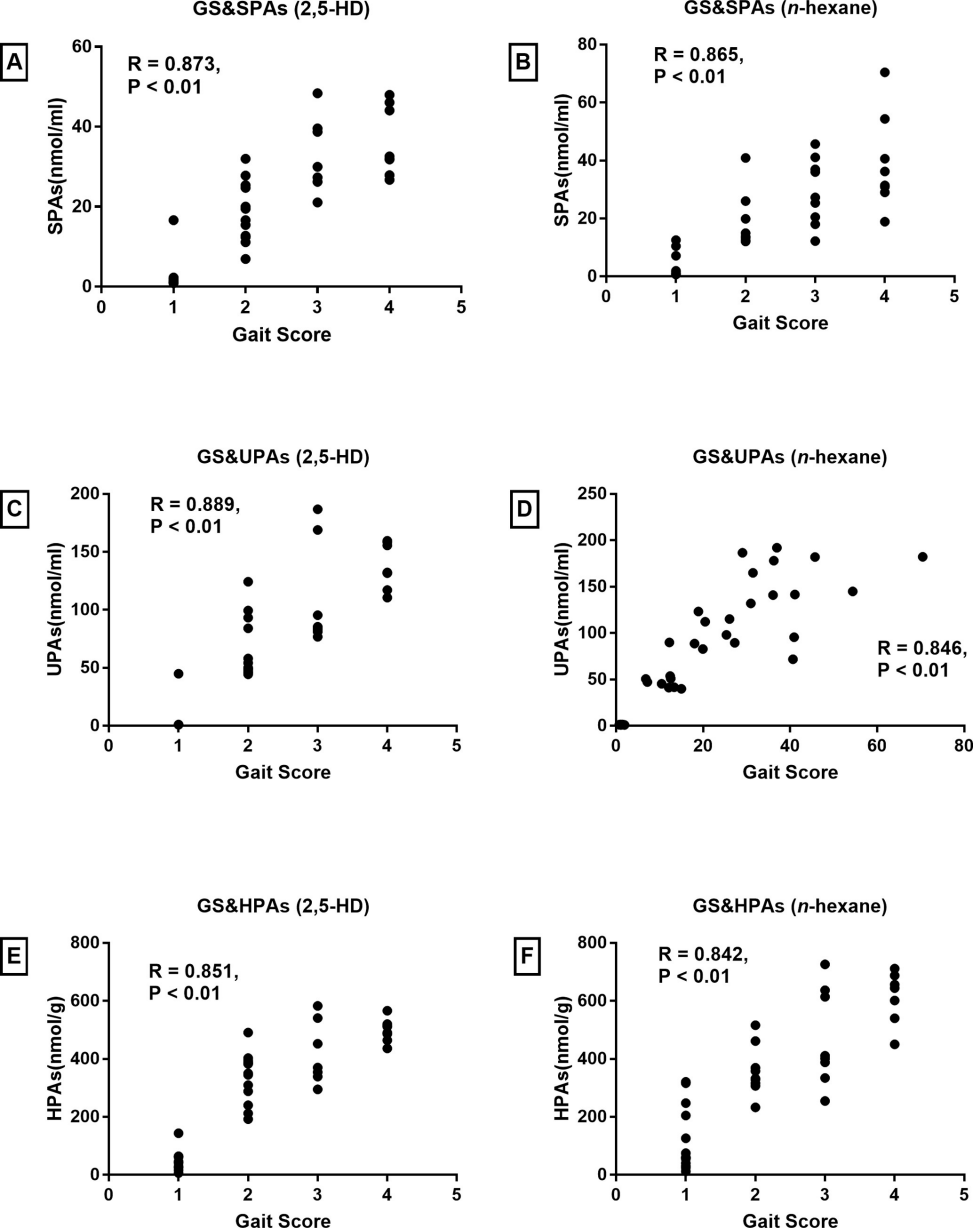
Correlation analysis. (A) Serum pyrrole adducts and gait score of rats treated with 2,5-HD. (B) Serum pyrrole adducts and gait score of rats treated with *n*-hexane. (C) Urine pyrrole adducts and gait score of rats treated with 2,5-HD. (D) Urine pyrrole adducts and gait score of rats treated with *n*-hexane. (E) Hair pyrrole adducts and gait score of rats treated with 2,5-HD. (F) Hair pyrrole adducts and gait score of rats treated with *n*-hexane.

Spearman’s correlation analysis between hair pyrrole adducts, urine, serum pyrrole adducts and gait score were conducted. As shown in Fig 3, serum, urine and hair pyrrole adducts all show highly relevant with the gait abnormality of rats. Then gait score was defined as Y considering its role as the golden standard assessing the impairment, and the pyrrole adducts in hair, urine and serum separately defined as X_1_, X_2_ and X_3_ to calculate the partial correlation coefficients (Partial R) (Table 5). Results showed that in both models, hair pyrrole adducts had a higher Partial R values than serum and urine pyrrole adducts.

**Table 5:** Multiply regression analysis and partial correlation.

	2,5-HD model	*n*-hexane model
PAs in serum (SPAs) & Gait Score	0.873^**^	0.865^**^
PAs in urine (UPAs) & Gait Score PAs in hair (HPAs) & Gait Score	0.889^**^ 0.851^**^	0.846^**^ 0.842^**^
Multiple R (Gait Score & BPAs, HPAs)	0.905^**^	0.891^**^
Partial R (Blood)	0.163^#^	0.187^#^
Partial R (Urine) Partial R (Hair)	0.325^*^ 0.397^*^	0.393^*^ 0.406^*^

^*^P<0.05,

^**^P<0.01,

^#^*P*>0.05.

## Discussion

Hair samples are more stable and convenient, showing accumulation statues. We hypothesized that pyrrole adducts in hair could serve as an effective biomarker. Although the accumulation of pyrrole adducts in hair have been reported for decades, no methods have been developed to quantify the concentration of pyrrole adducts. Moreover, no researches have analyzed the relationship between hair pyrrole adducts and neuropathy [23, 24].

Our researches before have already figured out methods of digestion and determining the pyrrole adducts in hair. The previous studies used different concentrations of 2,5-HD to soak the blank hair for 48 hours before the hair samples were digested. Then absorption values were measured and showed a dose-response relationship in the concentration of pyrrole adducts to exposed level [28]. However, in this study we aimed to use 2,5-HD to generated model to quantify the hair pyrrole adducts and then generate *n*-hexane model to confirm our findings.

Firstly, 2,5-HD induced PNS impairment models were generated. To avoid the direct contamination of 2,5-HD, we chose gavage 2,5-HD with saline vehicle to generate models. Moreover, dorsal hair was chosen, considering which might not easily contaminated by urine pyrrole adducts. Results showed pyrrole adducts accumulated in hair with a dose-response relationship among groups and highly relevant to neuropathy. Then, we doubted whether the phenomenon is consistent when rats exposed to original xenobiotic *n*-hexane. Though for *n*-hexane, inhalation ought to be the best choice, the aim of this model was to verify the data of 2,5-HD model ahead, finally gavage was chosen again to ensure the parallelism. The parallelism, as shown in our tables and figures, were in accordance with our original hypothesis. However, this study merely determined the pyrrole adducts at the endpoint, further study might conduct surveillance alongside the intoxication to get a comprehensive statue of pyrrole adducts accumulation.

Although this spectrophotometer method seems crude, but it is really practical considering pyrrole circle only originating form 2,5-HD in animal models. In this study, the hair samples of rats were shaved before treatment so the hair samples collected at the end of treatment were all newly grown. Thus, the accumulation of pyrrole adducts in hair is exactly in parallel with the administration to 2,5-HD or *n*-hexane exposure progress, representing a condition of alongside total exposure.

Two neuro-behavior indicators were used to evaluate the damage degree of PNS. Firstly, gait score were observed as the golden standard of paralysis with the score of 4; secondly, rota-rod were measured to earn a more objective assessment of animal motor condition as the auxiliary method. Therefore, we chose gait score instead of rota-rod latency to conduct spearman’s correlation analysis. Results showed pyrrole adducts in hair were highly relevant to the gait scores. This was the first time hair pyrrole adducts accumulation was reported positive relevant to the intoxication of *n*-hexane.

Commonly, serum and urine pyrrole adducts are effective biomarkers in occupational prevention of *n*-hexane intoxication. As showed in results, both pyrrole adducts also showed highly relevant to the gait scores, which were consistent with previous studies [21, 29, 30]. We intended to compare the representativeness of hair, serum and urine pyrrole adducts, therefore we performed multiple linear regression analysis. Results showed that the hair pyrrole adducts have higher partial correlation coefficients than serum and urine pyrrole adducts in both 2,5-HD and *n*-hexane models. The comparison suggested that hair pyrrole adducts might be a better biomarker. Though further human studies are needed to proof that.

Although 2,5-HD have been demonstrated to be the metabolite toxicant of *n*-hexane, there were some interesting comparison between the two models. Firstly, the weight changes of rats were slightly different, which mainly represented between the high dosage groups. At the endpoint of intoxication, rat of high dosage groups in n-hexane treated model have a higher average weight than those in 2,5-HD treated model., due to the low toxicity of *n*-hexane itself. Secondly, the pyrrole adducts in hair, serum and urine showed no differences between the same level dosage of two models, which was represented by the result that the transformation ratio of *n*-hexane to 2,5-HD was approximately 10 to 1. Thirdly, despite of the fact that abnormal neuro-behavior onset almost at the same time, the rats treated with 2,5-HD underwent paralyzed completely sooner than those treated with *n*-hexane. This delay in neuropathy progression of *n*-hexane treated groups might explained by the fact that 2,5-HD were generated from the metabolism in *n*-hexane treated models through CYP2E1 [8, 31]. Finally, the partial coefficients obtained in 2,5-HD model were smaller than those in the *n*-hexane model, which might be also explained by variance of individual biotransformation.

Surveillance of the hair pyrrole adducts could be of great help in the first prevention of occupational *n*-hexane intoxication. Although the mechanism of pyrrole adducts inducing PNS impairment stayed unclear, but there existed a possibility of long term monitoring hair pyrrole adducts to set practical biological exposure indices. Hence, our future study might be focus on the biological exposure indices of hair pyrrole adducts using rats chronically treated with a dosage of *n*-hexane at the no observed adverse effect level.

## Conclusions

This study successfully built peripheral nerve impairment models with 2,5-HD and *n*-hexane. The pyrrole adducts in hair could accumulate and their concentrations were highly relevant to the degree of PMS damage. Moreover, multiple regressive analysis showed hair pyrrole adducts have higher partial correlation coefficients than serum and urine pyrrole adducts. In summary, hair pyrrole adducts might be a promising biomarker to surveil the impairments of *n*-hexane induced neuropathy.

## Supporting information

S1 File Supporting information. The file includes tables of original data of weight changes (Table 1), gait scores (Table 2), rota-rod latencies (Table 3), pyrrole adducts at endpoint (Table 4). Table 5 was part of our previous study *in vitro* supporting our results. Fig 1 shows the pyrrole adducts in hair collected before the treatment. Fig 2 shows our surveillance of hair pyrrole adducts accumulation in another study which supporting our results.
(DOC)
