# Supplementary material for: Hair pyrrole adducts serve as biomarkers for peripheral nerve impairment induced by 2,5-hexanedione and n-hexane in rats
Source: PLoS One. 2018 Dec 31;13(12):e0209939. doi: 10.1371/journal.pone.0209939 (PMC6312332; doi:10.1371/journal.pone.0209939)
Supplement: S1 File — The file includes tables of original data of weight changes (Table 1), gait scores (Table 2), rota-rod latencies (Table 3), pyrrole adducts at endpoint (Table 4). Table 5 was part of our previous study in vitro supporting our results. Fig 1 shows the pyrrole adducts in hair collected before the treatment. Fig 2 shows our surveillance of hair pyrrole adducts accumulation in another study which supporting our results. (DOC) [file pone.0209939.s001.doc]

**Table 1. Weight changes**

|  | Control (2,5-HD) | | | | | | | | | |
| --- | --- | --- | --- | --- | --- | --- | --- | --- | --- | --- |
| Week | 1 | 2 | 3 | 4 | 5 | 6 | 7 | 8 | 9 | 10 |
| 0 | 216.3 | 208.5 | 216.5 | 215.9 | 246.5 | 206.4 | 208.4 | 205.2 | 208.7 | 210.1 |
| 1 | 275.2 | 256 | 264.5 | 262.2 | 264.4 | 253.9 | 261.7 | 268 | 262 | 262.4 |
| 2 | 309.7 | 279.8 | 300.1 | 294.4 | 297.1 | 277.2 | 290.4 | 304.6 | 288.6 | 296.9 |
| 3 | 352 | 298.3 | 345.5 | 329.3 | 337.6 | 305.7 | 332 | 345.5 | 330 | 326.1 |
| 4 | 369 | 319.1 | 356.3 | 339.3 | 357.9 | 321.3 | 355.8 | 359.5 | 350.6 | 333.5 |
| 5 | 387 | 316.9 | 354.6 | 342.8 | 354.2 | 323.6 | 354.2 | 352.4 | 345.9 | 328.4 |
| 6 | 399 | 343.9 | 392.6 | 375.3 | 364.4 | 353.6 | 375.9 | 363.5 | 358.3 | 342.1 |
| 7 | 420.1 | 353.1 | 402.2 | 395 | 403.5 | 354.2 | 390 | 392.7 | 369.7 | 382.5 |
| 8 | 417.3 | 360 | 416.8 | 390.6 | 404.3 | 361.5 | 398.1 | 405.4 | 378 | 381.3 |
|  | Low (2,5-HD) | | | | | | | | | |
| Week | 1 | 2 | 3 | 4 | 5 | 6 | 7 | 8 | 9 | 10 |
| 0 | 204.2 | 209.1 | 202.4 | 209.9 | 211 | 209.4 | 213.5 | 210.9 | 207 | 212 |
| 1 | 253.4 | 265.4 | 250 | 254.6 | 267.1 | 265 | 256 | 247.6 | 253 | 265.6 |
| 2 | 283.3 | 299 | 275 | 289 | 306.1 | 295.2 | 280.9 | 273.8 | 300.3 | 304.7 |
| 3 | 319 | 316.5 | 309.6 | 312.5 | 346 | 331.1 | 312.2 | 299.7 | 320 | 345.7 |
| 4 | 340.4 | 350.5 | 327.6 | 329 | 367.6 | 356.9 | 320.3 | 312.5 | 342.4 | 370.1 |
| 5 | 347.4 | 349.2 | 341.9 | 306.6 | 377.5 | 375.1 | 317 | 307.5 | 316.9 | 360 |
| 6 | 366.6 | 362.7 | 364.2 | 324.6 | 400.3 | 392.4 | 340.2 | 316.2 | 336.9 | 374.3 |
| 7 | 383.6 | 365.4 | 378.9 | 338.3 | 411.7 | 392.6 | 351.3 | 321.1 | 344 | 393 |
| 8 | 388.3 | 389.5 | 372.1 | 341.7 | 419.6 | 388.9 | 364.9 | 312.9 | 366.1 | 410.9 |
|  | Middle (2,5-HD) | | | | | | | | | |
| Week | 1 | 2 | 3 | 4 | 5 | 6 | 7 | 8 | 9 | 10 |
| 0 | 210.5 | 211 | 210.7 | 205.6 | 213 | 208.5 | 207.6 | 214 | 212.7 | 212.5 |
| 1 | 248.7 | 258.4 | 253.3 | 253.6 | 256.4 | 243.5 | 254 | 266.3 | 255.6 | 258.2 |
| 2 | 277.7 | 298.8 | 285.8 | 291.8 | 285.5 | 271.4 | 283.6 | 303.7 | 285.7 | 290.9 |
| 3 | 304 | 316.2 | 309.2 | 309.4 | 309.7 | 279.6 | 313 | 336.6 | 300 | 314.6 |
| 4 | 318.5 | 323.5 | 336.8 | 291.6 | 324.6 | 289 | 330.9 | 345.5 | 290 | 338 |
| 5 | 314.3 | 294.7 | 356.9 | 266 | 344.7 | 260.9 | 346.1 | 347.2 | 307.4 | 360 |
| 6 | 305 | 304.5 | 368 | 287.2 | 349 | 375.6 | 350 | 366.3 | 267.8 | 368.9 |
| 7 | 302.7 | 300.1 | 368.9 | 290.1 | 352.3 | 260.8 | 348 | 359 | 247.5 | 362.4 |
| 8 | 304 | 297.9 | 367.5 | 294.5 | 356.6 | 267.1 | 347 | 367.9 | 264.5 | 360.6 |
|  | High (2,5-HD) | | | | | | | | | |
| Week | 1 | 2 | 3 | 4 | 5 | 6 | 7 | 8 | 9 | 10 |
| 0 | 209.3 | 215.5 | 207.9 | 210.6 | 207.6 | 222 | 223.6 | 211.5 | 212 | 223.1 |
| 1 | 245.8 | 252.2 | 244.2 | 252.6 | 243.5 | 271.4 | 270 | 247 | 245.1 | 264 |
| 2 | 275.9 | 288.6 | 272.5 | 286.4 | 279 | 310 | 304 | 274.6 | 273.4 | 296.7 |
| 3 | 298 | 299.5 | 292.2 | 297.9 | 292.4 | 324.6 | 310.6 | 289 | 284 | 306 |
| 4 | 300.7 | 286.5 | 299.2 | 286.9 | 284.3 | 301.9 | 305.3 | 274.5 | 290.5 | 296.5 |
| 5 | 256 | 271.4 | 300.9 | 255.2 | 285.3 | 256.6 | 305.5 | 277 | 298 | 294.8 |
| 6 | 246.8 | 273.7 | 290.9 | 253.4 | 289.7 | 264.3 | 285.6 | 262.5 | 309.3 | 304.3 |
| 7 | 244.3 | 248.3 | 272.4 | 236.9 | 246.3 | 241.3 | 246.9 | 247.1 | 265.7 | 269.6 |
| 8 | 241.7 | 232.7 | 254 | 213.4 | 230.9 | 211.3 | 242 | 226.3 | 247.5 | 251.3 |

|  | Control (*n*-hexane) | | | | | | | | | |
| --- | --- | --- | --- | --- | --- | --- | --- | --- | --- | --- |
| Week | 1 | 2 | 3 | 4 | 5 | 6 | 7 | 8 | 9 | 10 |
| 0 | 226.3 | 238.5 | 226.5 | 225.6 | 240.1 | 215 | 222.5 | 221.5 | 210.5 | 220.5 |
| 1 | 242.9 | 253.1 | 249.1 | 243.5 | 242.59 | 229.1 | 254.9 | 244.4 | 253.3 | 252 |
| 2 | 274.8 | 278.6 | 284.4 | 249.5 | 269.4 | 258.3 | 279 | 275.1 | 274.2 | 276.1 |
| 3 | 290.1 | 285.7 | 289 | 247.2 | 296 | 274.8 | 298.1 | 300.1 | 294 | 289.3 |
| 4 | 317.4 | 308.8 | 320.9 | 269.9 | 311.6 | 295.7 | 328.3 | 320.7 | 321.5 | 307.2 |
| 5 | 317.4 | 311.3 | 331.3 | 279.9 | 318 | 310.9 | 349.5 | 346.7 | 344.3 | 331.5 |
| 6 | 340 | 321.3 | 351.2 | 303.9 | 336.8 | 324.8 | 370.8 | 359.8 | 356.6 | 344.6 |
| 7 | 355.8 | 336.5 | 365.1 | 334.3 | 352.1 | 342.2 | 381 | 371.9 | 383.6 | 346.8 |
| 8 | 350.7 | 340.2 | 362.7 | 347.3 | 367.8 | 353.9 | 392.3 | 384.8 | 388 | 369.7 |
| 9 | 387.6 | 367.6 | 400 | 368.8 | 393.9 | 370.8 | 423.6 | 397.2 | 402.9 | 379.6 |
| 10 | 381.5 | 375.7 | 410.6 | 373.3 | 391.1 | 374.7 | 417 | 406.8 | 409 | 387.6 |
|  | Low (*n*-hexane) | | | | | | | | | |
| Week | 1 | 2 | 3 | 4 | 5 | 6 | 7 | 8 | 9 | 10 |
| 0 | 214.5 | 219.5 | 212 | 229 | 221 | 219 | 223 | 210 | 217 | 222 |
| 1 | 292.7 | 279.9 | 272 | 291.5 | 303.4 | 258.9 | 236.9 | 244 | 269.4 | 239.1 |
| 2 | 303.1 | 307.1 | 310.2 | 344.7 | 285.4 | 265.4 | 287.1 | 290.7 | 294.4 | 274.5 |
| 3 | 309.5 | 323 | 327.7 | 332.3 | 296.6 | 279.3 | 300.4 | 325.3 | 316.4 | 316.1 |
| 4 | 310.3 | 338.2 | 330.8 | 320 | 314 | 283.4 | 301.4 | 332.1 | 324 | 325.5 |
| 5 | 306.4 | 340.4 | 346.6 | 344 | 329.7 | 300.9 | 304.7 | 340.7 | 343.2 | 292 |
| 6 | 321.6 | 352.8 | 362.8 | 364.8 | 344 | 314.1 | 317.8 | 337.7 | 359.3 | 312.6 |
| 7 | 326.1 | 354.3 | 384.2 | 380.6 | 348.9 | 321.1 | 318.7 | 379.3 | 342.3 | 344.8 |
| 8 | 330.2 | 363.8 | 399.9 | 395.7 | 354.3 | 339.5 | 328.7 | 386.3 | 357.7 | 377.3 |
| 9 | 334.7 | 372.3 | 412 | 411.9 | 354.1 | 346.2 | 336.4 | 396.4 | 378.1 | 404.3 |
| 10 | 327.5 | 340 | 407.3 | 410 | 350.7 | 368.5 | 328.8 | 392.6 | 357.4 | 404.5 |
|  | Middle (*n*-hexane) | | | | | | | | | |
| Week | 1 | 2 | 3 | 4 | 5 | 6 | 7 | 8 | 9 | 10 |
| 0 | 220.5 | 222.1 | 222 | 215 | 223 | 218 | 217 | 224 | 227 | 222 |
| 1 | 288 | 270 | 238 | 232 | 240.6 | 235.8 | 262 | 290 | 237.6 | 232.7 |
| 2 | 296.1 | 300.4 | 275.4 | 232.1 | 270 | 248.7 | 292.5 | 325.7 | 286.1 | 209.3 |
| 3 | 312 | 303.2 | 262.5 | 240.9 | 288.9 | 264.6 | 303.9 | 331 | 292.2 | 230.9 |
| 4 | 324.1 | 329.5 | 275 | 258.1 | 299.9 | 282.2 | 308.7 | 339.1 | 307 | 251.2 |
| 5 | 349.8 | 350 | 290.1 | 262.4 | 309 | 296.7 | 299.8 | 342.7 | 253.3 | 269.7 |
| 6 | 355.3 | 335.3 | 299.7 | 262.3 | 323.1 | 303 | 303.6 | 333.7 | 251.1 | 287.5 |
| 7 | 368.6 | 334.3 | 309.5 | 258.8 | 341.8 | 306.7 | 319.2 | 320.5 | 273.2 | 312.7 |
| 8 | 369.1 | 357 | 310.2 | 260.4 | 333.6 | 311.5 | 324.8 | 345.2 | 269.4 | 302.1 |
| 9 | 389 | 359 | 316.7 | 265 | 365.3 | 318.8 | 334.6 | 338.4 | 284.9 | 316.7 |
| 10 | 380.7 | 348 | 294.6 | 245.4 | 332.8 | 320.9 | 330.9 | 331.6 | 274.1 | 313 |
|  | High (*n*-hexane) | | | | | | | | | |
| Week | 1 | 2 | 3 | 4 | 5 | 6 | 7 | 8 | 9 | 10 |
| 0 | 219 | 225 | 217 | 220 | 217 | 232 | 233 | 222 | 222 | 233 |
| 1 | 225.3 | 233.3 | 227.9 | 218 | 261 | 218.9 | 247.1 | 229 | 236.5 | 242.6 |
| 2 | 266.8 | 202.1 | 260.1 | 218 | 270.9 | 229 | 262.5 | 280.1 | 292.9 | 256 |
| 3 | 274.3 | 213.8 | 245.4 | 223.4 | 284.7 | 232.9 | 289.3 | 272.9 | 294.8 | 250.4 |
| 4 | 264.6 | 240.7 | 257.3 | 217.6 | 284.2 | 214 | 281.1 | 284 | 317.6 | 235.8 |
| 5 | 290.9 | 267.9 | 262.9 | 233.9 | 303 | 244.7 | 304.5 | 299 | 340.9 | 265.5 |
| 6 | 295.7 | 350 | 272 | 243 | 319.7 | 269.1 | 312 | 270 | 306 | 268.7 |
| 7 | 364.5 | 306.9 | 285.2 | 252.5 | 336.1 | 286.2 | 324.1 | 291.9 | 317.2 | 276.8 |
| 8 | 314.2 | 301.3 | 292.8 | 253.1 | 347.7 | 273.8 | 331 | 348 | 384.1 | 274.9 |
| 9 | 298.6 | 285.5 | 291 | 240.6 | 328.5 | 264 | 309 | 331 | 370.8 | 263.3 |
| 10 | 312.4 | 299 | 297.8 | 204.1 | 330.1 | 216.6 | 314.4 | 342.6 | 360.1 | 232.2 |

**Table 2. Gait scores**

|  | Control (2,5-HD) | | | | | | | | | |
| --- | --- | --- | --- | --- | --- | --- | --- | --- | --- | --- |
| Week | 1 | 2 | 3 | 4 | 5 | 6 | 7 | 8 | 9 | 10 |
| 0 | 1 | 1 | 1 | 1 | 1 | 1 | 1 | 1 | 1 | 1 |
| 1 | 1 | 1 | 1 | 1 | 1 | 1 | 1 | 1 | 1 | 1 |
| 2 | 1 | 1 | 1 | 1 | 1 | 1 | 1 | 1 | 1 | 1 |
| 3 | 1 | 1 | 1 | 1 | 1 | 1 | 1 | 1 | 1 | 1 |
| 4 | 1 | 1 | 1 | 1 | 1 | 1 | 1 | 1 | 1 | 1 |
| 5 | 1 | 1 | 1 | 1 | 1 | 1 | 1 | 1 | 1 | 1 |
| 6 | 1 | 1 | 1 | 1 | 1 | 1 | 1 | 1 | 1 | 1 |
| 7 | 1 | 1 | 1 | 1 | 1 | 1 | 1 | 1 | 1 | 1 |
| 8 | 1 | 1 | 1 | 1 | 1 | 1 | 1 | 1 | 1 | 1 |
|  | Low (2,5-HD) | | | | | | | | | |
| Week | 1 | 2 | 3 | 4 | 5 | 6 | 7 | 8 | 9 | 10 |
| 0 | 1 | 1 | 1 | 1 | 1 | 1 | 1 | 1 | 1 | 1 |
| 1 | 1 | 1 | 1 | 1 | 1 | 1 | 1 | 1 | 1 | 1 |
| 2 | 1 | 1 | 1 | 1 | 1 | 1 | 1 | 1 | 1 | 1 |
| 3 | 1 | 1 | 1 | 1 | 1 | 1 | 1 | 1 | 1 | 1 |
| 4 | 1 | 1 | 1 | 1 | 1 | 1 | 1 | 1 | 1 | 1 |
| 5 | 1 | 1 | 1 | 1 | 1 | 1 | 1 | 1 | 1 | 1 |
| 6 | 1 | 1 | 1 | 1 | 2 | 1 | 2 | 1 | 1 | 1 |
| 7 | 1 | 1 | 2 | 1 | 2 | 2 | 2 | 1 | 1 | 1 |
| 8 | 2 | 2 | 2 | 2 | 1 | 2 | 2 | 2 | 2 | 2 |
|  | Middle (2,5-HD) | | | | | | | | | |
| Week | 1 | 2 | 3 | 4 | 5 | 6 | 7 | 8 | 9 | 10 |
| 0 | 1 | 1 | 1 | 1 | 1 | 1 | 1 | 1 | 1 | 1 |
| 1 | 1 | 1 | 1 | 1 | 1 | 1 | 1 | 1 | 1 | 1 |
| 2 | 1 | 1 | 1 | 1 | 1 | 1 | 1 | 1 | 1 | 1 |
| 3 | 1 | 1 | 1 | 1 | 1 | 1 | 1 | 1 | 1 | 1 |
| 4 | 1 | 1 | 1 | 1 | 1 | 1 | 1 | 2 | 1 | 1 |
| 5 | 2 | 2 | 2 | 2 | 2 | 2 | 2 | 2 | 2 | 2 |
| 6 | 2 | 2 | 2 | 2 | 2 | 2 | 2 | 2 | 2 | 2 |
| 7 | 2 | 2 | 2 | 2 | 2 | 2 | 2 | 2 | 2 | 2 |
| 8 | 3 | 2 | 3 | 2 | 3 | 3 | 3 | 2 | 3 | 2 |
|  | High (2,5-HD) | | | | | | | | | |
| Week | 1 | 2 | 3 | 4 | 5 | 6 | 7 | 8 | 9 | 10 |
| 0 | 1 | 1 | 1 | 1 | 1 | 1 | 1 | 1 | 1 | 1 |
| 1 | 1 | 1 | 1 | 1 | 1 | 1 | 1 | 1 | 1 | 1 |
| 2 | 1 | 1 | 1 | 1 | 1 | 1 | 1 | 1 | 1 | 1 |
| 3 | 1 | 1 | 1 | 1 | 1 | 1 | 1 | 2 | 1 | 1 |
| 4 | 2 | 2 | 2 | 2 | 2 | 2 | 2 | 2 | 2 | 2 |
| 5 | 2 | 3 | 2 | 2 | 2 | 3 | 3 | 3 | 3 | 2 |
| 6 | 3 | 3 | 3 | 3 | 3 | 3 | 3 | 3 | 3 | 2 |
| 7 | 3 | 3 | 3 | 3 | 3 | 4 | 3 | 3 | 3 | 3 |
| 8 | 4 | 4 | 3 | 4 | 4 | 4 | 4 | 4 | 3 | 4 |

| Control (*n*-hexane) | | | | | | | | | | |
| --- | --- | --- | --- | --- | --- | --- | --- | --- | --- | --- |
| week | 1 | 2 | 3 | 4 | 5 | 6 | 7 | 8 | 9 | 10 |
| 0 | 1 | 1 | 1 | 1 | 1 | 1 | 1 | 1 | 1 | 1 |
| 1 | 1 | 1 | 1 | 1 | 1 | 1 | 1 | 1 | 1 | 1 |
| 2 | 1 | 1 | 1 | 1 | 1 | 1 | 1 | 1 | 1 | 1 |
| 3 | 1 | 1 | 1 | 1 | 1 | 1 | 1 | 1 | 1 | 1 |
| 4 | 1 | 1 | 1 | 1 | 1 | 1 | 1 | 1 | 1 | 1 |
| 5 | 1 | 1 | 1 | 1 | 1 | 1 | 1 | 1 | 1 | 1 |
| 6 | 1 | 1 | 1 | 1 | 1 | 1 | 1 | 1 | 1 | 1 |
| 7 | 1 | 1 | 1 | 1 | 1 | 1 | 1 | 1 | 1 | 1 |
| 8 | 1 | 1 | 1 | 1 | 1 | 1 | 1 | 1 | 1 | 1 |
| 9 | 1 | 1 | 1 | 1 | 1 | 1 | 1 | 1 | 1 | 1 |
| 10 | 1 | 1 | 1 | 1 | 1 | 1 | 1 | 1 | 1 | 1 |
| Low (*n*-hexane) | | | | | | | | | | |
| week | 1 | 2 | 3 | 4 | 5 | 6 | 7 | 8 | 9 | 10 |
| 0 | 1 | 1 | 1 | 1 | 1 | 1 | 1 | 1 | 1 | 1 |
| 1 | 1 | 1 | 1 | 1 | 1 | 1 | 1 | 1 | 1 | 1 |
| 2 | 1 | 1 | 1 | 1 | 1 | 1 | 1 | 1 | 1 | 1 |
| 3 | 1 | 1 | 1 | 1 | 1 | 1 | 1 | 1 | 1 | 1 |
| 4 | 1 | 1 | 1 | 1 | 1 | 1 | 1 | 1 | 1 | 1 |
| 5 | 1 | 1 | 1 | 1 | 1 | 1 | 1 | 1 | 1 | 1 |
| 6 | 1 | 1 | 1 | 1 | 1 | 1 | 1 | 1 | 1 | 1 |
| 7 | 1 | 1 | 1 | 1 | 1 | 1 | 1 | 1 | 1 | 1 |
| 8 | 1 | 1 | 1 | 1 | 1 | 1 | 1 | 1 | 1 | 1 |
| 9 | 1 | 2 | 1 | 1 | 2 | 1 | 1 | 1 | 1 | 2 |
| 10 | 1 | 1 | 1 | 2 | 2 | 2 | 2 | 1 | 1 | 2 |
| Middle (*n*-hexane) | | | | | | | | | | |
| week | 1 | 2 | 3 | 4 | 5 | 6 | 7 | 8 | 9 | 10 |
| 0 | 1 | 1 | 1 | 1 | 1 | 1 | 1 | 1 | 1 | 1 |
| 1 | 1 | 1 | 1 | 1 | 1 | 1 | 1 | 1 | 1 | 1 |
| 2 | 1 | 1 | 1 | 1 | 1 | 1 | 1 | 1 | 1 | 1 |
| 3 | 1 | 1 | 1 | 1 | 1 | 1 | 1 | 1 | 1 | 1 |
| 4 | 1 | 1 | 1 | 1 | 1 | 1 | 1 | 1 | 1 | 1 |
| 5 | 2 | 1 | 1 | 1 | 1 | 1 | 1 | 1 | 1 | 1 |
| 6 | 2 | 1 | 1 | 1 | 1 | 1 | 1 | 1 | 1 | 1 |
| 7 | 2 | 2 | 2 | 2 | 1 | 1 | 2 | 1 | 2 | 1 |
| 8 | 2 | 3 | 2 | 2 | 1 | 2 | 2 | 2 | 2 | 2 |
| 9 | 3 | 3 | 3 | 2 | 2 | 2 | 2 | 2 | 3 | 2 |
| 10 | 3 | 4 | 3 | 2 | 3 | 3 | 2 | 2 | 4 | 3 |
| High (*n*-hexane) | | | | | | | | | | |
| week | 1 | 2 | 3 | 4 | 5 | 6 | 7 | 8 | 9 | 10 |
| 0 | 1 | 1 | 1 | 1 | 1 | 1 | 1 | 1 | 1 | 1 |
| 1 | 1 | 1 | 1 | 1 | 1 | 1 | 1 | 1 | 1 | 1 |
| 2 | 2 | 1 | 1 | 1 | 1 | 1 | 1 | 1 | 1 | 1 |
| 3 | 2 | 1 | 1 | 1 | 2 | 1 | 1 | 1 | 1 | 2 |
| 4 | 2 | 1 | 1 | 2 | 2 | 1 | 1 | 1 | 1 | 2 |
| 5 | 2 | 2 | 1 | 2 | 2 | 1 | 1 | 1 | 1 | 3 |
| 6 | 3 | 2 | 1 | 2 | 2 | 1 | 2 | 1 | 1 | 3 |
| 7 | 4 | 3 | 1 | 2 | 3 | 2 | 3 | 2 | 3 | 3 |
| 8 | 4 | 3 | 2 | 2 | 3 | 2 | 3 | 3 | 4 | 3 |
| 9 | 4 | 4 | 3 | 3 | 3 | 3 | 3 | 3 | 4 | 3 |
| 10 | 4 | 4 | 3 | 4 | 4 | 4 | 4 | 4 | 4 | 3 |

**Table 3. Rota-rod latencies**

|  | Control (2,5-HD) | | | | | | | | | |
| --- | --- | --- | --- | --- | --- | --- | --- | --- | --- | --- |
| Week | 1 | 2 | 3 | 4 | 5 | 6 | 7 | 8 | 9 | 10 |
| 0 | 57 | 96 | 65 | 85 | 97 | 82 | 69 | 97 | 117 | 77 |
| 1 | 65 | 110 | 74 | 97 | 111 | 94 | 79 | 111 | 133 | 88 |
| 2 | 85 | 138 | 99 | 124 | 131 | 140 | 100 | 172 | 150 | 101 |
| 3 | 99 | 179 | 112 | 121 | 120 | 148 | 109 | 133 | 160 | 130 |
| 4 | 100 | 91 | 140 | 150 | 128 | 142 | 105 | 139 | 200 | 91 |
| 5 | 98 | 97 | 111 | 146 | 103 | 142 | 118 | 137 | 200 | 122 |
| 6 | 85 | 99 | 94 | 139 | 107 | 84 | 117 | 138 | 200 | 90 |
| 7 | 86 | 110 | 96 | 130 | 111 | 95 | 120 | 144 | 200 | 105 |
| 8 | 90 | 116 | 90 | 125 | 116 | 100 | 111 | 130 | 180 | 106 |
|  | Low (2,5-HD) | | | | | | | | | |
| Week | 1 | 2 | 3 | 4 | 5 | 6 | 7 | 8 | 9 | 10 |
| 0 | 85 | 67 | 64 | 69 | 81 | 84 | 100 | 86 | 115 | 71 |
| 1 | 97 | 74 | 68 | 61 | 100 | 60 | 55 | 70 | 19 | 120 |
| 2 | 91 | 99 | 73 | 93 | 113 | 73 | 81 | 100 | 99 | 95 |
| 3 | 124 | 141 | 93 | 110 | 127 | 85 | 107 | 89 | 136 | 119 |
| 4 | 77 | 91 | 144 | 130 | 89 | 71 | 128 | 117 | 124 | 77 |
| 5 | 109 | 147 | 75 | 76 | 72 | 77 | 98 | 115 | 97 | 79 |
| 6 | 110 | 102 | 64 | 69 | 81 | 84 | 100 | 86 | 115 | 71 |
| 7 | 116 | 100 | 60 | 65 | 75 | 80 | 95 | 80 | 110 | 69 |
| 8 | 102 | 95 | 58 | 60 | 70 | 75 | 90 | 78 | 108 | 61 |
|  | Middle (2,5-HD) | | | | | | | | | |
| Week | 1 | 2 | 3 | 4 | 5 | 6 | 7 | 8 | 9 | 10 |
| 0 | 67 | 64 | 69 | 81 | 84 | 100 | 123 | 86 | 71 | 67 |
| 1 | 97 | 60 | 92 | 82 | 56 | 70 | 66 | 111 | 84 | 80 |
| 2 | 92 | 54 | 53 | 71 | 59 | 97 | 61 | 94 | 85 | 89 |
| 3 | 99 | 61 | 82 | 55 | 68 | 117 | 75 | 79 | 94 | 83 |
| 4 | 122 | 81 | 94 | 74 | 83 | 96 | 69 | 70 | 90 | 82 |
| 5 | 129 | 71 | 77 | 61 | 74 | 98 | 94 | 99 | 106 | 86 |
| 6 | 67 | 65 | 75 | 78 | 70 | 78 | 83 | 72 | 68 | 67 |
| 7 | 65 | 60 | 61 | 71 | 65 | 73 | 74 | 65 | 62 | 61 |
| 8 | 60 | 55 | 55 | 59 | 59 | 54 | 53 | 60 | 55 | 53 |
|  | High (2,5-HD) | | | | | | | | | |
| Week | 1 | 2 | 3 | 4 | 5 | 6 | 7 | 8 | 9 | 10 |
| 0 | 60 | 80 | 91 | 70 | 80 | 84 | 76 | 88 | 84 | 80 |
| 1 | 54 | 75 | 91 | 64 | 72 | 74 | 66 | 60 | 95 | 69 |
| 2 | 50 | 75 | 80 | 50 | 92 | 64 | 44 | 67 | 64 | 85 |
| 3 | 58 | 87 | 97 | 55 | 98 | 81 | 73 | 63 | 70 | 74 |
| 4 | 21 | 95 | 71 | 54 | 67 | 50 | 66 | 53 | 73 | 51 |
| 5 | 20 | 83 | 82 | 40 | 39 | 50 | 49 | 20 | 36 | 25 |
| 6 | 21 | 60 | 61 | 20 | 24 | 30 | 36 | 15 | 30 | 16 |
| 7 | 19 | 44 | 42 | 13 | 14 | 15 | 26 | 9 | 25 | 5 |
| 8 | 10 | 24 | 25 | 5 | 5 | 5 | 15 | 5 | 16 | 3 |

|  | Control (*n*-hexane) | | | | | | | | | |
| --- | --- | --- | --- | --- | --- | --- | --- | --- | --- | --- |
| Week | 1 | 2 | 3 | 4 | 5 | 6 | 7 | 8 | 9 | 10 |
| 0 | 118 | 104 | 107 | 109 | 111 | 107 | 118 | 97 | 118 | 125 |
| 1 | 120 | 116 | 111 | 108 | 135 | 136 | 140 | 125 | 123 | 135 |
| 2 | 137 | 130 | 123 | 112 | 155 | 173 | 175 | 148 | 149 | 157 |
| 3 | 125 | 112 | 116 | 109 | 145 | 167 | 165 | 150 | 140 | 150 |
| 4 | 119 | 100 | 110 | 105 | 140 | 160 | 170 | 135 | 132 | 140 |
| 5 | 111 | 90 | 105 | 100 | 124 | 130 | 170 | 125 | 122 | 136 |
| 6 | 102 | 73 | 93 | 96 | 112 | 89 | 174 | 84 | 119 | 127 |
| 7 | 105 | 86 | 91 | 105 | 106 | 85 | 150 | 96 | 117 | 130 |
| 8 | 99 | 80 | 91 | 110 | 90 | 79 | 146 | 110 | 110 | 124 |
| 9 | 99 | 90 | 92 | 118 | 87 | 75 | 135 | 112 | 108 | 125 |
| 10 | 96 | 87 | 89 | 127 | 77 | 60 | 132 | 113 | 109 | 122 |
|  | Low (*n*-hexane) | | | | | | | | | |
| Week | 1 | 2 | 3 | 4 | 5 | 6 | 7 | 8 | 9 | 10 |
| 0 | 87 | 93 | 115 | 103 | 110 | 127 | 119 | 121 | 106 | 95 |
| 1 | 105 | 100 | 125 | 104 | 110 | 126 | 119 | 130 | 115 | 100 |
| 2 | 118 | 105 | 138 | 105 | 110 | 125 | 120 | 135 | 125 | 109 |
| 3 | 105 | 110 | 120 | 100 | 113 | 114 | 119 | 130 | 115 | 107 |
| 4 | 99 | 111 | 119 | 95 | 116 | 105 | 121 | 125 | 100 | 106 |
| 5 | 86 | 100 | 109 | 97 | 140 | 108 | 123 | 119 | 95 | 95 |
| 6 | 76 | 82 | 96 | 90 | 133 | 89 | 123 | 117 | 74 | 86 |
| 7 | 84 | 99 | 100 | 82 | 113 | 104 | 119 | 106 | 66 | 88 |
| 8 | 80 | 111 | 110 | 77 | 120 | 111 | 111 | 92 | 70 | 70 |
| 9 | 87 | 111 | 116 | 69 | 111 | 116 | 120 | 90 | 71 | 75 |
| 10 | 85 | 123 | 115 | 63 | 103 | 121 | 114 | 81 | 65 | 66 |
|  | Middle (*n*-hexane) | | | | | | | | | |
| Week | 1 | 2 | 3 | 4 | 5 | 6 | 7 | 8 | 9 | 10 |
| 0 | 103 | 93 | 122 | 125 | 90 | 130 | 92 | 140 | 102 | 105 |
| 1 | 113 | 85 | 120 | 114 | 140 | 120 | 112 | 126 | 99 | 113 |
| 2 | 124 | 80 | 126 | 97 | 162 | 116 | 117 | 113 | 94 | 112 |
| 3 | 111 | 75 | 119 | 95 | 130 | 104 | 106 | 99 | 89 | 110 |
| 4 | 100 | 71 | 110 | 90 | 110 | 95 | 90 | 90 | 80 | 100 |
| 5 | 99 | 72 | 99 | 84 | 105 | 94 | 85 | 85 | 87 | 96 |
| 6 | 91 | 62 | 97 | 86 | 95 | 82 | 72 | 71 | 88 | 92 |
| 7 | 92 | 63 | 87 | 89 | 84 | 79 | 73 | 73 | 88 | 95 |
| 8 | 93 | 60 | 77 | 70 | 77 | 70 | 70 | 79 | 82 | 80 |
| 9 | 96 | 59 | 66 | 67 | 69 | 64 | 69 | 75 | 89 | 76 |
| 10 | 94 | 57 | 57 | 56 | 51 | 51 | 66 | 78 | 99 | 71 |
|  | High (*n*-hexane) | | | | | | | | | |
| Week | 1 | 2 | 3 | 4 | 5 | 6 | 7 | 8 | 9 | 10 |
| 0 | 107 | 102 | 107 | 109 | 102 | 125 | 103 | 99 | 121 | 126 |
| 1 | 99 | 105 | 99 | 100 | 99 | 130 | 106 | 96 | 115 | 114 |
| 2 | 95 | 103 | 79 | 75 | 86 | 144 | 96 | 86 | 89 | 95 |
| 3 | 76 | 100 | 80 | 69 | 84 | 126 | 99 | 89 | 97 | 84 |
| 4 | 66 | 99 | 90 | 66 | 77 | 99 | 96 | 85 | 99 | 77 |
| 5 | 45 | 85 | 95 | 56 | 69 | 84 | 94 | 89 | 65 | 56 |
| 6 | 23 | 72 | 100 | 44 | 64 | 74 | 94 | 87 | 41 | 39 |
| 7 | 17 | 56 | 95 | 21 | 51 | 64 | 74 | 67 | 25 | 36 |
| 8 | 11 | 46 | 76 | 15 | 42 | 56 | 66 | 40 | 12 | 40 |
| 9 | 5 | 23 | 55 | 12 | 21 | 24 | 24 | 31 | 10 | 46 |
| 10 | 2 | 10 | 40 | 6 | 11 | 7 | 9 | 20 | 6 | 45 |

**Table 4. Pyrrole adducts at endpoint**

| 2,5-HD | 1 | 2 | 3 | 4 | 5 | 6 | 7 | 8 | 9 | 10 |
| --- | --- | --- | --- | --- | --- | --- | --- | --- | --- | --- |
| Serum C | 1.3 | 1.5 | 1.0 | 0.9 | 1.2 | 0.9 | 1.9 | 2.3 | 1.2 | 1.2 |
| Serum L | 11.1 | 6.9 | 12.7 | 12.6 | 16.6 | 16.6 | 19.5 | 15.4 | 12.4 | 20.0 |
| Serum M | 1.1 | 1.6 | 39.6 | 24.7 | 21.1 | 30.0 | 27.3 | 32.0 | 38.7 | 27.8 |
| Serum H | 31.8 | 32.6 | 27.3 | 48.0 | 32.0 | 44.1 | 46.1 | 26.7 | 48.4 | 27.9 |
|  | | | | | | | | | | |
| 2,5-HD | 1 | 2 | 3 | 4 | 5 | 6 | 7 | 8 | 9 | 10 |
| Urine C | 1.1 | 0.9 | 1.4 | 0.7 | 1.1 | 1.0 | 1.8 | 1.6 | 1.1 | 1.0 |
| Urine L | 45.7 | 45.8 | 44.5 | 49.2 | 45.0 | 54.2 | 50.1 | 48.2 | 46.5 | 58.0 |
| Urine M | 1.3 | 1.3 | 84.6 | 93.2 | 95.4 | 76.8 | 81.0 | 84.1 | 85.4 | 124.3 |
| Urine H | 131.8 | 131.7 | 168.9 | 159.5 | 158.4 | 155.6 | 132.2 | 117.1 | 186.8 | 110.6 |
|  | | | | | | | | | | |
| 2,5-HD | 1 | 2 | 3 | 4 | 5 | 6 | 7 | 8 | 9 | 10 |
| Hair C | 25.2 | 6.3 | 6.3 | 42.0 | 46.1 | 29.3 | 40.9 | 16.1 | 19.9 | 25.2 |
| Hair L | 240.1 | 394.1 | 388.0 | 350.1 | 143.8 | 212.2 | 288.0 | 383.0 | 403.0 | 192.2 |
| Hair M | 64.2 | 61.5 | 339.0 | 385.1 | 294.8 | 354.0 | 355.9 | 490.9 | 541.2 | 345.2 |
| Hair H | 490.4 | 566.4 | 370.5 | 464.4 | 511.8 | 514.4 | 486.3 | 520.0 | 583.3 | 436.1 |

| *n*-hexane | 1 | 2 | 3 | 4 | 5 | 6 | 7 | 8 | 9 | 10 |
| --- | --- | --- | --- | --- | --- | --- | --- | --- | --- | --- |
| Serum C | 0.9 | 1.1 | 1.4 | 1.1 | 1.9 | 0.7 | 1.6 | 1.9 | 1.0 | 1.3 |
| Serum L | 7.2 | 10.5 | 13.8 | 13.4 | 15.0 | 12.1 | 7.2 | 6.8 | 12.5 | 12.4 |
| Serum M | 20.5 | 27.3 | 18.9 | 40.9 | 18.0 | 12.2 | 19.9 | 26.1 | 40.7 | 25.4 |
| Serum H | 29.1 | 41.1 | 45.7 | 31.4 | 70.5 | 36.1 | 36.9 | 31.0 | 54.4 | 36.2 |
|  | | | | | | | | | | |
| *n*-hexane | 1 | 2 | 3 | 4 | 5 | 6 | 7 | 8 | 9 | 10 |
| Hair C | 12.6 | 25.2 | 24.2 | 59.9 | 60.7 | 40.3 | 30.8 | 75.2 | 43.8 | 55.9 |
| Hair L | 126.1 | 316.1 | 247.8 | 329.2 | 516.2 | 461.9 | 316.1 | 205.0 | 320.7 | 369.9 |
| Hair M | 334.2 | 601.4 | 401.9 | 307.2 | 410.8 | 388.2 | 358.3 | 233.3 | 540.6 | 726.3 |
| Hair H | 450.2 | 255.0 | 725.3 | 332.2 | 644.3 | 614.5 | 636.8 | 656.3 | 711.4 | 687.8 |
|  | | | | | | | | | | |
| *n*-hexane | 1 | 2 | 3 | 4 | 5 | 6 | 7 | 8 | 9 | 10 |
| Urine C | 1.3 | 0.9 | 1.3 | 1.3 | 1.0 | 1.1 | 1.1 | 0.7 | 1.1 | 1.2 |
| Urine L | 47.3 | 45.2 | 42.4 | 41.5 | 40.0 | 50.6 | 49.3 | 41.2 | 50.8 | 53.7 |
| Urine M | 112.2 | 89.5 | 123.3 | 95.5 | 88.6 | 89.9 | 82.8 | 71.8 | 115.1 | 98.1 |
| Urine H | 186.6 | 141.6 | 145.0 | 164.9 | 182.3 | 140.9 | 192.0 | 132.0 | 182.0 | 178.0 |

**Table 5. In vitro test**

Support information to line 293:

We used different concentrations of 2,5-HD to soak the hair samle from rats and human who have not been exposed to 2,5-HD or n-hexane. 2,5-HD solution were prepared using saine and soak the hair with the ratio of 1g to 10 ml for 48 hours. Then, hair samples were collected and determined asforesaid methods, and the concertration of pyrrole adducts in hair showd dose-response ship and no difference between models.

|  | Rat hair model | | | | Human hair model | | | |
| --- | --- | --- | --- | --- | --- | --- | --- | --- |
|  | OD value | PAs（nmol/ml） | Protein（mg/ml） | Justified（nmol/mg.pro） | OD value | PAs（nmol/ml） | Protein（mg/ml） | Justified（nmol/mg.pro） |
|  |  |  |  |  |  |  |  |  |
| Control | 0.063 | 0.21±0.04 | 3.57±0.01 | 0.06±0.01 | 0.062 | 0.28±0.05 | 4.33±0.02 | 0.07±0.01 |
| 10mg/ml | 0.147 | 17.78±5.73 | 3.75±0.02 | 4.73±0.02** | 0.152 | 18.9±3.30 | 3.27±0.01 | 5.79±0.01** |
| 20mg/ml | 0.231 | 35.44±5.34 | 3.61±0.02 | 9.82±1.48** | 0.238 | 36.9±6.06 | 3.59±0.03 | 10.26±1.69** |
| 30mg/ml | 0.327 | 55.27±1.74 | 3.58±0.05 | 15.44±0.49** | 0.318 | 53.6±0.60 | 3.13±0.02 | 16.25±1.05** |

Compare with control, *** P*< 0.01.

**Figure 1. Pyrrole adducts in hair before treated**





|  | Control | Low | Middle | High |
| --- | --- | --- | --- | --- |
| PAs (nmol/g) | 32.3 ± 6.2 | 34.1 ± 2.7 | 35.9 ± 1.0 | 28.8 ± 5.4 |





|  | Control | Low | Middle | High |
| --- | --- | --- | --- | --- |
| PAs (nmol/g) | 40.6 ± 4.0 | 39.7 ± 5.4 | 42.3 ± 4.1 | 44.7 ± 3.1 |

**Figure 2. Hair pyrrole adducts changes**

Support information to the comment 1 of reviewer 1:

We did surveille hair pyrrole adducts in another experiments. This is a graph showing changes of pyrrole adducts in hair of rats exposed to n-hexane. Hair sampes were collected every two weeks and determined due to the slow growth of hair. There were differences since the second week, sooner than the outcome of neuro-impairments.
